# Enhanced sweet pepper yield through high-intensity artificial lighting and optimized plant density in high-latitude winter production

**DOI:** 10.7717/peerj.21491

**Published:** 2026-06-30

**Authors:** Christina Stadler

**Affiliations:** Agricultural University of Iceland, Reykjavík, Iceland

**Keywords:** Additional lighting, *Capsicum annuum*, Intracanopy lighting, Interlighting, Supplemental lighting, Top lighting

## Abstract

In Iceland, extremely low natural light levels during winter make greenhouse crop production largely dependent on supplemental lighting. This study evaluated the interactive effects of plant density and lighting configuration on yield and fruit quality of sweet pepper (*Capsicum annuum* cv. Ferrari) under controlled cabinet conditions. Plants were cultivated at two densities under high-pressure sodium (HPS) lamps, using either top lighting (TL) alone or in combination with interlighting (IL). Under low natural light level, marketable yield tended to increase with both higher light intensity and plant density, primarily due to a greater number of fruits per unit area, while average fruit weight remained relatively stable. Under higher natural light level, the effects of plant density and lighting were minimal. The combination of TL and IL slightly increased unmarketable fruit due to blossom-end rot and light-induced damage. These results suggest that higher plant densities, when combined with adequate supplemental lighting, may enhance yield during periods of limited natural light in high-latitude greenhouses.

## Introduction

The extremely low levels of natural light during winter are the primary limiting factor for winter greenhouse production in Iceland. As a result, supplemental lighting is essential to enable year-round vegetable cultivation (Chen et al., 2024; Fanwoua et al., 2019). Winter production using artificial lighting could reduce the need for imports from southern regions during the winter months. As a general rule, a 1% increase in light intensity can result in a 0.7–1.0% increase in yield for fruiting vegetables (Marcelis et al., 2006).

Traditionally, supplemental lighting in greenhouses has been provided from above the canopy (top lighting), which limits light availability to the lower leaves. However, lower canopy leaves remain photosynthetically active, and their light limitation can reduce overall plant productivity (Hovi-Pekkanen & Tahvonen, 2008). To improve light distribution within the canopy, studies have examined the use of interlighting—placing lights within the crop rows—in greenhouse-grown tomato (*Solanum lycopersicum* L.) (Gunnlaugsson & Adalsteinsson, 2006), cucumber (*Cucumis sativus* L.) (Pettersen, Torre & Gislerød, 2010), and sweet pepper (*Capsicum annuum* L.) (Hovi-Pekkanen, Näkkilä & Tahvonen, 2006) under northern climatic conditions. In the Netherlands, the narrow aisle widths in commercial greenhouses restrict the use of high-pressure sodium (HPS) lamps for interlighting, leading to growing interest in light-emitting diodes (LEDs) for this purpose while HPS lamps are retained for top lighting (Trouwborst et al., 2011).

In addition to lighting, plant density is a crucial factor influencing crop performance in greenhouse systems. Appropriate plant spacing ensures proper growth and development of plants and maximizes yield potential (Dalvi et al., 2024; Sharma & Kumar, 2017). However, plant spacing also affects competition for resources and light interception, which may influence both yield and quality (Abriham, 2024). Plant density strongly determines the utilization of light interception, mainly through effects on leaf area index (Nasto, Balliu & Zeka, 2009). The need for appropriate plant densities to maximize production per unit area by utilizing the available space is especially important in protected cultivation (Spehia et al., 2014). Several studies have indicated an increase in total yield when plant density is increased and the increase in yield with higher plant density was a result of increased number of fruits per unit area. For example, Motsenbocker (1996) reported that in pepperoncini peppers, higher densities reduced biomass and yield per plant but increased total yield and fruit number per square meter. The influence of plant density on fruit quality is also well documented. Nasto, Balliu & Zeka (2009) observed that increasing plant density (2.6, 3.7, 4.8, 8.3, 11.1 plants m^−2^) resulted in a nearly linear increase in total harvested pepper yield due to a greater number of fruits, although marketable yield per fruit decreased significantly at higher densities. These findings suggest that adjusting plant density according to lighting regimes may be an effective strategy to optimize light interception and crop yield.

Overall, both light distribution and plant density are key determinants of productivity in greenhouse-grown vegetables. However, research examining their combined effects—particularly in sweet pepper—remains limited. In this context, the positioning of luminaires may play a critical role in optimizing light distribution, especially under varying plant density regimes. Therefore, the objectives of this study were to investigate whether (1) plant density should be adjusted according to light intensity, and (2) the positioning of the lighting, in conjunction with plant density, affects the yield and outer fruit quality of sweet pepper.

## Materials and Methods

### Plant material and experimental set-up

**Figure 1:**
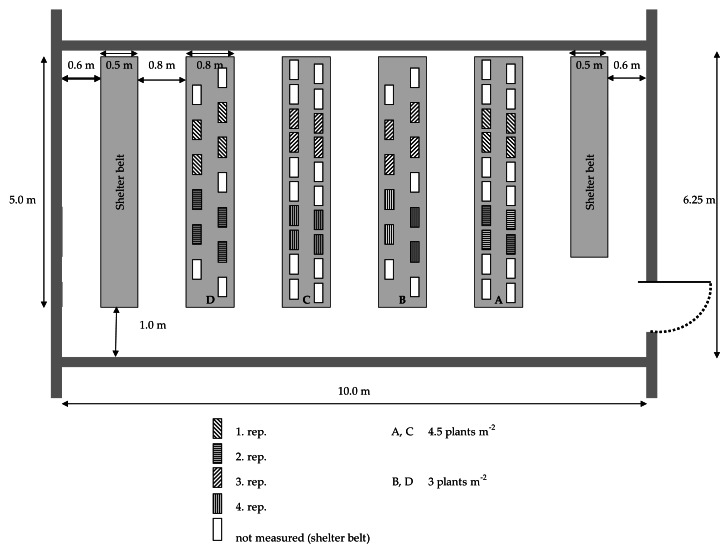
Experimental set-up of cabinets.

An experiment with sweet pepper (*Capsicum annuum* L., cv. Ferrari) was conducted in the experimental greenhouse at Reykir, south Iceland (64°0.178′N, 21°10.639′W). At the time of the experiment, the greenhouse was owned by the Agricultural University of Iceland; it is now owned by the Sudurland College. Seeds were sown on 22 August 2008 in Grodan^®^ stone wool plugs. The seedlings were transplanted to stone wool cubes on 16 September, and on 20 October, pairs of plants were transplanted into 11-liter Bato buckets (40 cm × 25 cm × 15 cm) filled with pumice stones. The buckets were arranged in double rows in four separate greenhouse cabinets (6.25 m × 10.0 m each). Within each cabinet, four central planting beds (5.0 m × 0.8 m; designated A, B, C, and D; Fig. 1) were used for the experiment, while one additional bed at each end served as shelter beds. Plant density was varied across the beds: one of the two inner beds (B or C) and one of the two outer beds (A or D) in each cabinet were assigned a low density of 3.0 plants m^−2^ (13 pots per bed; B and D). The remaining two beds were assigned a high density of 4.5 plants m^−2^ (20 pots per bed; A and C). Each plant was trained to two stems and grown until 27 July 2009.

### Lighting regimes

Plants were cultivated under high-pressure sodium (HPS) lamps using either top lighting (TL) alone or a combination of top lighting and interlighting (IL). Four different lighting regimes were applied, with each greenhouse cabinet assigned one regime:

(1) TL 160 W m^−2^ (TL 160),
(2) TL 120 W m^−2^ + IL 120 W m^−2^ (TL 120 + IL 120),
(3) TL 240 W m^−2^ (TL 240),
(4) TL 160 W m^−2^ + IL 120 W m^−2^ (TL 160 + IL 120)

HPS lamps for top lighting (600 W bulbs; SuperAgro, Gavita, Norway) were mounted horizontally above the plant canopy, positioned 4 m above ground level in the aisles between the planting beds. Interlighting was provided using 250 W HPS bulbs (Gavita, Norway). Initially, these interlighting fixtures were installed 0.25–0.50 m above the canopy, depending on plant height. On 17 March 2009, they were repositioned between the plant rows, approximately 0.90 m above ground and aligned with the center axis of each bed.

Lighting was operated for a maximum of 18 h per day. The lights were automatically turned off when the incoming natural radiation exceeded a predefined set-point, based on empirical experience (from February to mid-March: lamps were turned off when natural radiation exceeded 300 W m^−2^, from mid-March to July: the threshold was increased to 400 W m^−2^).

**Figure 2:**
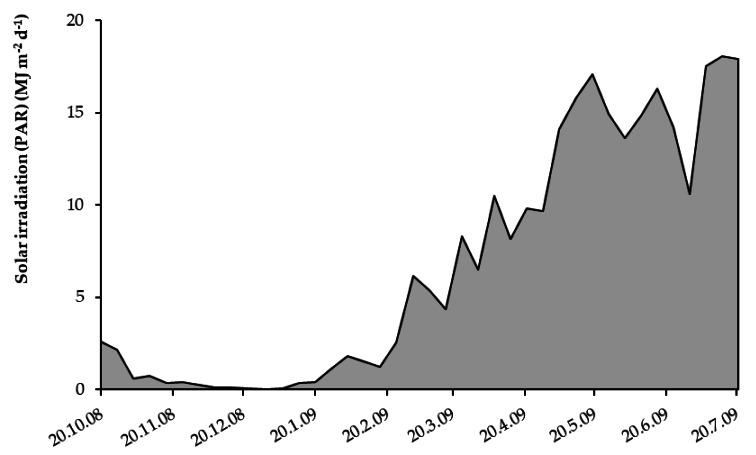
Solar irradiation (PAR) at Reykir (64° 0.178′N, 21°10.639′W) during the cropping season of sweet pepper in 2008/2009. Solar irradiation was measured every day and values for one week were cumulated.

In Iceland, solar irradiation is extremely low during winter. From October 2008 through the end of February 2009, daily photosynthetically active radiation (PAR) levels remained below 5 MJ m^−2^ d^−1^ (low natural light level) (Fig. 2). As day length increased, natural radiation gradually rose, reaching 8–11 MJ m^−2^ d^−1^ (high natural light level) by mid-April. All radiation values were automatically recorded at the experimental site.

As expected, the different lighting regimes influenced both the vertical distribution of illuminance and the surrounding air temperature within the crop canopy. These parameters were measured under diffuse light conditions from the base of the plant (0 m) up to the canopy top (2.0 m), at 0.25 m intervals, and at representative horizontal positions within the beds. Shortly after transplanting, the measured illuminance levels at canopy height were as follows: 31 klux under TL 160 + IL 120, 27 klux under TL 120 + IL 120, 19 klux under TL 240, 14 klux under TL 160. Throughout the cultivation period, illuminance at 1.5–2.0 m height (upper canopy) was approximately 10 klux higher under TL 240 compared to TL 120 + IL 120. However, in the mid to lower canopy (0.5–1.0 m), TL 240 provided 2–4 klux less light than the TL 120 + IL 120 treatment. Regarding temperature, TL 240 resulted in air temperatures that were 1–4 °C lower at 1.0–1.5 m height compared to the TL 120 + IL 120 treatment. At other heights, the interlighting system generally maintained temperatures up to 1 °C lower.

### Growth conditions

Greenhouse air temperature was maintained at 22–23 °C during the day and 18–19 °C at night. However, as noted in the previous section, temperature around the plants varied depending on the position and type of lighting used.

Carbon dioxide (CO_2_) enrichment was applied to support photosynthesis and yield. CO_2_ concentration was maintained at 800 ppm when ventilation was closed and reduced to ambient levels (approximately 400 ppm) when ventilation was triggered at temperatures above 24 °C. Due to differences in ventilation frequency among the lighting regimes, variations in CO_2_ levels between cabinets cannot be excluded and may have influenced plant growth and yield. A misting system was activated whenever relative humidity (RH) dropped below 70%.

Sweet pepper plants received standard nutrient solution *via* drip irrigation, with three tubes per bucket. The electrical conductivity (EC) of the solution was maintained between 1.8 and 2.5 dS m^−1^, and pH was kept between 5.2 and 6.5, adjusted according to the EC of the drainage and plant growth stage. While fertilizer composition remained constant across treatments, irrigation frequency and duration were adjusted according to plant age and developmental stage. Plant protection was primarily based on biological control using beneficial organisms, and insecticides were applied only when necessary. No fungicides were used during the experiment.

### Sampling and measurements

In each planting bed, two groups of eight plants (a total of 16 plants per bed) located centrally were designated as subplots for measurements. The remaining plants along the edges of the beds served as a shelter belt. Throughout the growing period, green and red fruits (with >50% of the surface area red) were regularly harvested from the subplot plants. Harvested fruits were classified into the following categories: total yield, number of fruits, marketable fruits (>100 g), and non-marketable fruits (including fruits weighing <100 g, fruits with blossom-end rot, and fruits damaged by lighting).

### Statistical analyses

Average values for each planting bed were calculated from the two subplot samples (16 plants per bed). For each plant density, two composite samples were created—one from an inner bed and one from an outer bed—and these were analysed separately. Inner and outer beds were not pooled, as inner beds consistently produced higher yields than outer beds, likely due to more favourable microclimatic conditions.

Thus, for each cabinet, two mixed samples (inner and outer bed) were treated as independent replicates in the statistical analysis, even though both originated from the same cabinet. While lighting regimes were not replicated across cabinets (*i.e.,* each lighting treatment was applied in only one cabinet), the error component attributed to cabinet-specific effects was expected to be minimal. The author acknowledges potential differences in air temperature and CO_2_ concentration between cabinets, as these are inherently linked to the lighting regimes. However, efforts were made to minimize such differences by adjusting temperature and CO_2_ set points to be as similar as possible across all treatments.

All statistical analyses were performed using SAS Version 9.4 (SAS Institute Inc., Cary, North Carolina, USA). Differences in yield were analysed with analysis of variance (ANOVA) to assess the effects of plant density, bed position (inner *vs.* outer), cabinet/light intensity, and the interactions between light intensity and bed position as well as light intensity and plant density. Statistical significance was determined using Tukey–Kramer HSD tests at *p* = 0.05.

To further evaluate the adjusted effect estimates, multivariate regression analyses were conducted using the PROC GLM procedure. Non-significant interaction terms were removed sequentially, beginning with the least significant, and the model was refined iteratively until the final model with the highest overall significance was obtained.

## Results

**Figure 3:**
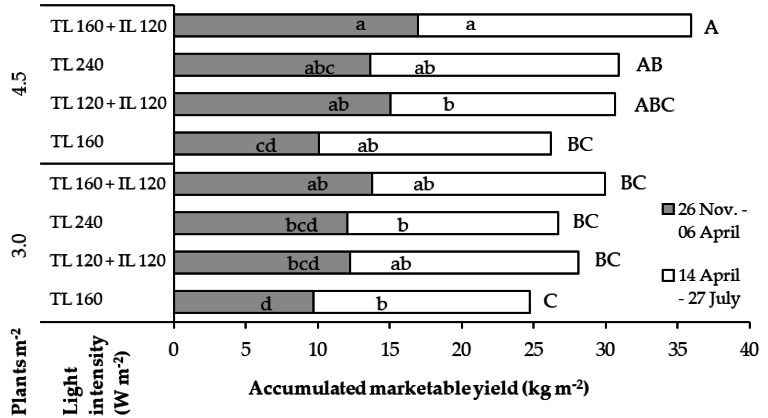
Accumulated marketable yield of sweet pepper (variety Ferrari) at different lighting regimes (top lighting: TL, interlighting: IL) and plant densities (3.0 and 4.5 plants m^−2^) at low natural light level (26 Nov. –6 April) and at high natural light level (14 April–27 July). Numbers after TL and IL represent the light intensity in W m^−2^. Values sharing the same small letter indicate that there are no significant differences at low/high natural light level and values sharing the same capital letter indicate that there are no significant differences in total yield at the end of the harvest period (HSD, *p* = 0.05).

At the lowest light intensity (TL 160), the accumulated marketable yield was not significantly affected by plant density (approximately 25 kg m^−2^ for both 3.0 and 4.5 plants m^−2^) (Fig. 3). In contrast, at the highest light intensity (TL 160 + IL 120), marketable yield was significantly higher at the higher plant density of 4.5 plants m^−2^ compared to 3.0 plants m^−2^, with yields of approximately 36 kg m^−2^ and 30 kg m^−2^, respectively.

The accumulated marketable yield over the entire harvest period was influenced primarily by plant density, with light intensity and bed position (inner *vs.* outer) also having smaller, yet detectable effects (Table 1(a)). The interaction between light intensity and bed position as well as the interaction between light intensity and plant density were both small and statistically non-significant, indicating good consistency in treatment effects across the different cabinet positions. Therefore, the observed yield increase at the higher plant density can be attributed mainly to the increased light intensity, as both density levels were tested within each cabinet. However, it should be noted that the potential influence of air temperature and CO_2_ concentration, which may have varied slightly between cabinets, cannot be entirely excluded.

**Table 1:** Analysis of variance of main effects and interactions of accumulated marketable yield during the whole harvest period (a), from 26 Nov.–06 April (b) and from 14 April–27 July (c).

		**Whole harvest period (a)**		**26 Nov.–06 April (b)**		**14 April–27 July (c)**	
	**DF**	**MS**	**Pr > F**	**MS**	**Pr > F**	**MS**	**Pr > F**
PD	1	50.27	0.0045	15.80	0.0017	9.75	0.0354
LI	3	37.93	0.0047	20.87	0.0006	3.52	0.1279
P of B	1	40.77	0.0065	21.16	0.0010	3.20	0.1483
LI x P of B	3	6.49	0.0966	1.17	0.1027	2.50	0.1985
LI x PD	3	3.94	0.1889	1.53	0.0690	2.05	0.2496
Error	4	1.51		0.29		1.00	

**Notes.**

PD Plant density LI Light intensity P of B Position of bed

The increase in marketable yield of sweet pepper due to higher light intensity was most pronounced during the period of low natural light availability (Table 1(b)). During this time, outdoor temperatures were low, allowing for minimal ventilation and the maintenance of elevated CO_2_ levels, which may have contributed to enhanced growth and yield.

However, from mid-April onward, as outdoor temperatures increased and ventilation was required more frequently, the accumulated yield under high natural light conditions was less affected by plant density, although the effect remained statistically significant (Table 1(c)). During this later period, none of the other main effects or their interactions showed significant influence on yield, suggesting that the benefit of increased plant density under high light intensity was primarily limited to periods of low natural light.

**Figure 4:**
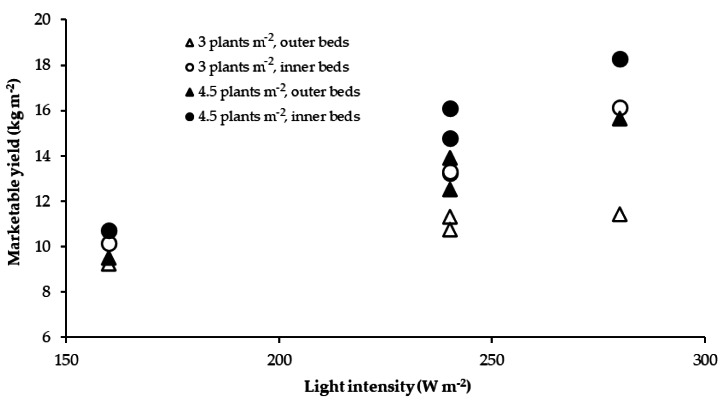
Relationship between accumulated marketable yield of sweet pepper and light intensity (installed capacity) during the time from 26 Nov.–06 April.

The relationship between accumulated marketable yield and total light intensity (combined top lighting and interlighting) was best explained by a statistical model that included the effects of plant density, bed position, and the interaction between light intensity and plant density. This model showed a strong fit, with an r^2^ value of 0.94 (Table 2). The model clearly demonstrates the yield advantage of higher plant density, particularly at higher light intensities, as indicated by the steeper slope of the yield response curve for the 4.5 plants m^−2^ treatment (Fig. 4).

**Table 2:** Statistics of the relationship between accumulated marketable yield of sweet pepper and light intensity during 26 Nov.–06 April.

**Source**	**DF**	**SS**	**MS**	**F Value**	Pr > F
Model	4	101.86	25.47	40.18	< 0.0001
Error	11	6.97	0.63		
Corrected total	15	108.83			
	**R-Square**	**Coeff Var**	**Root MSE**	**Yield mean**
	0.94	6.15	0.80	12.95

**Main effects + interactions**								
**GLM procedure**	**DF**	**MS**	**Pr > F**		**Estimate**	**Standard error**	**T Value**	**Pr > —t—**
Intercept					2.1679	1.5247	1.42	0.1828
PD	1	15.80	0.0004	1	3.2571	2.1378	1.52	0.1558
P of B	1	21.16	0.0001	1	−2.3000	0.3981	−5.78	0.0001
LI x PD	2	32.45	<0.0001	1	0.0334	0.0065	5.17	0.0003
LI x PD				2	0.0562	0.0065	8.70	<0.0001

**Notes.**

PD Plant density P of B Position of bed LI Light intensity

Marketable yield accounted for 84–88% of the total yield across treatments (Table 3). Increased light intensity did not affect marketable yield and had no effect on fruit size. However, the combination of top lighting and interlighting (TL 120 + IL 120 and TL 160 + IL 120) was associated with an increased incidence of blossom-end rot and fruit damage caused by lighting, whereas small fruits were not affected. Increasing plant density did not significantly affect fruit size.

**Table 3:** Proportion of marketable and unmarketable yield of sweet pepper at different plant densities and at top lighting (TL) alone and at top lighting (TL) together with interlighting (IL).

**Plants m** ^−2^	**Lighting regime**	**Marketable yield (%)**	**Unmarketable yield (%)**		
			**Too little weight**	**Blossom end rot**	**Damage from lighting**
4.5	TL 160	87^abc^	10^a^	2^b^	0^b^
	TL 120 + IL 120	84^cd^	8^a^	5^a^	3^a^
	TL 240	88^a^	7^a^	3^ab^	0^b^
	TL 160 + IL 120	84^d^	8^a^	5^a^	2^a^
3	TL 160	87^ab^	8^a^	3^ab^	0^b^
	TL 120 + IL 120	85^abcd^	7^a^	5^a^	2^a^
	TL 240	87^ab^	8^a^	4^ab^	0^b^
	TL 160 + IL 120	84^bcd^	6^a^	5^a^	3^a^

**Notes.**

Values with the same superscript within columns do not differ significantly at *p* = 0.05 in the analysis of variance.

Average fruit weight was not significantly affected by the lighting regimes. Red fruits weighed on average 147–157 g, while green fruits averaged 127–131 g.

## Discussion

The results of this study suggest that light intensity and plant density interact under the specific conditions of the experiment to influence sweet pepper yield, particularly under low natural light conditions during winter months. Marketable yield tended to increase with higher total light intensity, corresponding to a 0.5–0.8% yield increase per 1% increase in artificial light intensity, in line with the previously reported linear relationship between intercepted radiation and fruit yield in fruiting vegetables (Marcelis et al., 2006). Under high supplemental lighting, yield increased at the higher plant density, aligning with research in cucumber where a 1% increase in plant density (from 1.4 to 2.2 plants m^−2^) resulted in a 0.5% yield improvement without compromising fruit quality (Heuvelink et al., 2006). While these results indicate that light availability remains a key limiting factor for productivity during periods of low solar radiation, the lack of replication at the cabinet level means that these effects should be interpreted as trends observed under controlled conditions rather than as broadly generalizable causal relationships.

The increase in yield under high light intensity was primarily associated with a greater number of fruits per unit area rather than increased individual fruit size, as average fruit weight remained stable across treatments. Therefore, under the experimental conditions, yield gains did not appear to compromise fruit quality. This pattern mirrors previous observations, where higher light intensity stimulated fruit set rather than fruit enlargement (Heuvelink et al., 2006). This agrees with findings by Hwang et al. (2023), who observed similar trade-offs between fruit number and size when stem or plant density was increased. Similar patterns have been documented in studies manipulating stem number: Yoon et al. (2021) observed that increasing from two to three stems per plant decreased fruit fresh weight and fruit dry weight by 12–17% at multiple harvest times, emphasizing that increases in fruit number often occur at the expense of individual fruit mass. Such results highlight that yield gains in controlled environments often arise from greater reproductive sink formation rather than changes in source strength per fruit.

Plant density further modulated this light-yield relationship. Higher density (4.5 plants m^−2^) tended to improve total and marketable yields only under high light intensity (TL 160 + IL 120), suggesting that dense canopies can only be fully exploited when adequate irradiance supports canopy photosynthesis. Under low light (TL 160), yield differences between densities were minimal, implying that excessive shading under insufficient radiation can constrain potential benefits of higher planting density. This observation is consistent with 3D ray-tracing simulations showing that yield gains from higher density occur only when supplemental lighting compensates for self-shading (Hwang et al., 2023). Similarly, previous work in sweet pepper and cucumber indicated that while fruit yield per plant decreases with closer spacing, total yield per area increases up to an optimal density (Aminifard et al., 2010; Spehia et al., 2014; Dalvi et al., 2024).

Seasonal variation also influenced the magnitude of light and density effects. During winter and early spring, when natural solar radiation is minimal, both plant density and light intensity tended to affect marketable yield, whereas their influence was less pronounced under high summer irradiance (Table 1). Comparable seasonal trends have been reported in tomato, cucumber, and sweet pepper (Hovi-Pekkanen, Näkkilä & Tahvonen, 2006; Hovi-Pekkanen & Tahvonen, 2008; Näkkilä, Hovi-Pekkanen & Tahvonen, 2007; Hwang et al., 2023), where yield responses to lighting were strongest under low natural light level. Moreover, greenhouse climate factors such as CO_2_ concentration and air temperature may interact with light intensity. During winter, reduced ventilation allows higher CO_2_ retention, supporting photosynthetic efficiency (Hovi-Pekkanen, Näkkilä & Tahvonen, 2006). Although efforts were made to maintain uniform temperature across treatments, minor differences in canopy temperature (up to 4 °C cooler under high top-light intensity) may have influenced transpiration and carbon assimilation balance, contributing marginally to yield variation.

These results reinforce the importance of tailoring supplemental lighting strategies to seasonal conditions. As natural light level increases, the proportional contribution of artificial lighting diminishes, reducing yield differences between lighting regimes (Hovi-Pekkanen, Näkkilä & Tahvonen, 2006). Hence, concentrating supplemental lighting during periods of low solar radiation can help maintain yield stability while minimizing energy costs. In practice, determining optimal planting density must account for seasonal light availability. For example, in The Netherlands, sweet pepper production commonly uses a two- or three-stem system, with an optimal stem density of 6–6.5 stems m^−2^ under good light conditions (Spaargaren, 2001), emphasizing that target density should be adjusted according to light environment.

The positive yield trends observed with combined top lighting and interlighting treatments further highlight the role of within-canopy light distribution in enhancing productivity. Interlighting improved light penetration and photosynthetic activity in the lower canopy, reducing light limitation for shaded leaves (Chen et al., 2024; Goo & Park, 2024). This effect is particularly beneficial under dense plantings, where light interception at lower canopy levels is often suboptimal. However, the slight increase in blossom-end rot and light burns observed under interlighting suggests that excessive localized irradiance or heat may induce physiological stress. Similar effects were reported by Hovi-Pekkanen, Näkkilä & Tahvonen (2006) in sweet pepper, whereas in cucumber and tomato, interlighting improved fruit quality and shelf life (Hovi-Pekkanen & Tahvonen, 2008; Schouten et al., 2024). These species-specific responses underscore the need for optimizing lamp positioning and spectral composition to maximize yield benefits while minimizing fruit disorders.

Overall, the present study underscores the interactive effects of light intensity, plant density, and season on sweet pepper productivity under the specific cabinet conditions tested. Under high irradiance, dense canopies capture more photons per ground area, resulting in greater total biomass and fruit number (Hwang et al., 2023). In contrast, under limited light, increased density may reduce individual plant performance without compensatory gains at the crop level. These results suggest that balancing plant density and light availability—both natural and artificial—is essential for achieving sustainable yield increases in greenhouse sweet pepper cultivation.

## Conclusion

The results of this study suggested that, under the specific conditions of this experiment, the strategic use of supplemental lighting in combination with optimal plant density may enhance the yield and quality of sweet pepper in greenhouse environments during periods of low natural light level. However, due to the lack of replication at the cabinet level, these effects should be interpreted with caution and cannot be generalized beyond these controlled conditions. The potential benefits of increased lighting need to be considered in relation to energy costs and possible impacts on fruit quality. Future experiments could focus primarily on the low-light winter period, without the need to extend trials into spring and summer when natural light is more abundant. Additionally, further research on optimizing lighting intensity, spectral quality, and plant density would be valuable for enhancing productivity and sustainability in high-latitude greenhouse production.

##  Supplemental Information

10.7717/peerj.21491/supp-1 Supplemental Information 1 Yield data of sweet pepper

10.7717/peerj.21491/supp-2 Supplemental Information 2 Data average—weight of sweet pepper

## Additional Information and Declarations

### Data Availability

The following information was supplied regarding data availability: The raw measurements are available in the Supplemental Files.
